# Placental Nutrient Transporters and Maternal Fatty Acids in SGA, AGA, and LGA Newborns From Mothers With and Without Obesity

**DOI:** 10.3389/fcell.2022.822527

**Published:** 2022-03-25

**Authors:** Juan-Antonio Garcia-Santillan, Maria-Luisa Lazo-de-la-Vega-Monroy, Gloria-Celina Rodriguez-Saldaña, Miguel-Angel Solis-Barbosa, Maria-Angelica Corona-Figueroa, Martha-Isabel Gonzalez-Dominguez, Hector-Manuel Gomez-Zapata, Juan-Manuel Malacara, Gloria Barbosa-Sabanero

**Affiliations:** ^1^ Medical Sciences Department, Health Sciences Division, University of Guanajuato, Campus Leon, Guanajuato, Mexico; ^2^ Universidad de la Cienega del Estado de Michoacan de Ocampo, Trayectoria de Ingenieria en Nanotecnologia, Sahuayo, Mexico; ^3^ UMAE No. 48 IMSS, Gynecology and Obstetrics, Research Department, Leon, Guanajuato, Mexico

**Keywords:** placenta, maternal obesity, nutrient transporters, fatty acids, birth weight

## Abstract

Adverse environmental factors in early life result in fetal metabolic programming and increased risk of adult diseases. Birth weight is an indirect marker of the intrauterine environment, modulated by nutrient availability and placental transport capacity. However, studies of placental transporters in idiopathic birth weight alterations and in maternal obesity in relation to neonatal metabolic outcomes are scarce. We aimed to analyze the placental nutrient transporter protein expression in small (SGA, n = 14), adequate (AGA, n = 18), and large (LGA n = 10) gestational age term for newborns from healthy or obese mothers (LGA-OB, n = 9) and their association with maternal fatty acids, metabolic status, placental triglycerides, and neonatal outcomes. The transporter expression was determined by Western blot. The fatty acid profile was evaluated by gas chromatography, and placental triglycerides were quantified by an enzymatic colorimetric method. GLUT1 was higher in LGA and lower in SGA and positively correlated with maternal HbA1c and placental weight (PW). SNAT2 was lower in SGA, while SNAT4 was lower in LGA-OB. FATP1 was lower in SGA and higher in LGA. SNAT4 correlated negatively and FATP1 correlated positively with the PW and birth anthropometry (BA). Placental triglycerides were higher in LGA and LGA-OB and correlated with pregestational BMI, maternal insulin, and BA. Maternal docosahexaenoic acid (DHA) was higher in SGA, specifically in male placentas, correlating negatively with maternal triglycerides, PW, cord glucose, and abdominal perimeter. Palmitic acid (PA) correlated positively with FATP4 and cord insulin, linoleic acid correlated negatively with PA and maternal cholesterol, and arachidonic acid correlated inversely with maternal TG and directly with FATP4. Our study highlights the importance of placental programming in birth weight both in healthy and obese pregnancies.

## Introduction

Birth weight is an indirect marker of intrauterine growth and nutrition. Birth weight alterations have been associated with adverse perinatal effects and risk of disease in adulthood. Both extremes of birth weight, either SGA (small gestational age) or LGA (large gestational age), have an increased risk of developing diabetes mellitus type 2, hypertension, obesity, insulin resistance, prostate cancer, and cardiovascular diseases in adult life (Das and Sysyn, 2004; Monasta et al., 2010; Kruger et al., 2017; Fall and Kumaran, 2019; Sharma et al., 2020).

Birth weight is influenced by several maternal factors such as genes, nutritional and hormonal state, and the capacity of nutrient transport through the placenta. Thus, availability of nutrients such as glucose, amino acids, and fatty acids from the mother is determinant for adequate fetal growth (Jones et al., 2007; Hayward et al., 2016).

Glucose is the main cellular energy substrate for the fetus, which contributes directly with intrauterine development. Glucose transport *via* the placenta occurs by facilitated diffusion, trough concentration gradient from the mother to fetus, or by the glucose transporter (GLUT) family (Jansson et al., 1993; Prendergast et al., 1999). GLUTs are proteins composed of 12 transmembrane domains, of approximately 500 amino acid residues in total, which are encoded by the *SLC2* gene family. The main isoforms that have been detected in the human placenta are GLUT1, GLUT3, GLUT4, and GLUT9, having each one a specific function during glucose transport and exchange (Arnott et al., 1994; Illsley, 2000; Bibee et al., 2011; Mueckler and Thorens, 2013). The protein expression of GLUT1 in the placenta has been positively associated with the birth weight of newborns from women with obesity without type 2 diabetes mellitus (Acosta et al., 2015). Decreased GLUT1 protein has also been reported in the placenta of women with preeclampsia (Lüscher et al., 2017). On the other hand, the protein expression of GLUT3 is higher in primary trophoblast cultures of placentas from IURG pregnancy (Janzen et al., 2013) and lower in placentas from women with gestational diabetes mellitus (Zhang et al., 2016).

Similar to glucose, amino acids have specific functions for adequate fetal development as they are precursors for structure proteins and non-proteic substances such as polyamines, nitric oxide, neurotransmitters, and purine and pyrimidine nucleotides (Grillo et al., 2008). Placental amino acid transfer to the fetus is mediated by two coordinated systems, termed system A and L. System A transports nonessential neutral amino acids by a sodium-dependent mechanism, comprising the protein transporters SNAT1, SNAT2, and SNAT4, encoded by the *SLC38A* gene family. System L consists of a heterotrimeric complex, with the L-amino acid transporter LAT1 or LAT2, together with a heavy chain 4F2hc/CD98 protein, and exchanges essential amino acids in exchange for nonessential ones, in a sodium-independent fashion (Vaughan et al., 2017; Dumolt et al., 2021). Proteins of both systems have been detected in the human placenta, mainly in the microvillous membranes facing the maternal side (Vaughan et al., 2017). System A activity and the SNAT protein expression in the human placenta, but not in the LAT expression or system L, have been found decreased in IUGR (Shibata et al., 2008; Chen et al., 2015) and correlated with the birth weight in normal and obese mothers (Jansson et al., 2013; Gaccioli et al., 2015).

Long-chain fatty acids are essential for the fetal growth, serving as eicosanoid precursors, forming cellular plasmatic membrane, and mediating the genic expression. Furthermore, 50% of the neural tissue is composed by lipids, most of which are long-chain fatty acids. An increment of the total amount of fatty acids is seen throughout pregnancy, mainly long-chain polyunsaturated fatty acids (LC-PUFAs), such as docosahexaenoic acid 22:6n-3 (DHA) and arachidonic acid 20:4n-6 (AA), which increase 23 and 51%, respectively (Al et al., 2000). The human body is not capable of producing fatty acids with a double bond in carbons 3 and 6, which is why the intake of essential fatty acids into diet, such as linoleic acid (18:2 n-6) and α-linolenic acid (18:3 n-3), is required, in order to ensure adequate concentrations of their corresponding LC-PUFA derivatives (Haggarty, 2004; Duttaroy and Basak, 2020).

Fatty acid transport into the cell occurs by passive diffusion and through protein binding. Placental fatty acid transporters include FAT/CD36 (fatty acid translocase), FABPpm (plasma membrane fatty acid-binding protein), p-FABPpm (fatty acid-binding protein in the plasma membrane of the placenta), and FATP-1 to-6 (fatty acid-transporting proteins). Particularly, FATPs, a group of transmembrane proteins encoded by the *SLC27A* gene family, are located both in the MVM and in the BM, and their overexpression has been related to an increase in fatty acid internalization, presumably long-chain fatty acids, even at low concentrations (Larqué et al., 2011; Duttaroy and Basak, 2020). FATP1 (Coe et al., 1999) and FATP4 (Hall et al., 2005) are the only transporters that also exhibit the acyl-CoA-synthetase activity, which converts the transported fatty acids into acyl-CoA metabolites, preventing their efflux. It has been suggested that this transport is crucial for increased beta-oxidation of fatty acids (Duttaroy and Basak, 2020). A positive correlation between FATP1 and FATP4 placental mRNA levels with maternal plasma DHA levels and with umbilical cord phospholipid DHA has been found (Larqué et al., 2006). Also, a higher placental protein expression of FATP1 and FATP4 has been observed in mid-gestation in obese ewes compared to lean ewes (Zhu et al., 2010). Likewise, the FATP4 mRNA and protein expression are lower in placentas of women with obesity (Mishra et al., 2012).

Most previous investigations are focused on the study of nutrient transporters’ functions in the human placenta with pregnancy complications, such as preeclampsia, and gestational diabetes mellitus. Therefore, the aim of the present study was to evaluate the expression of glucose, amino acid, and fatty acid transporters in placentas of women without pregnancy pathologies, with or without obesity, and their relationship with maternal fatty acids and metabolic status. We hypothesize that there is a differential protein expression of the nutrient transporters in placentas of SGA, AGA, and LGA neonates, as well as a relationship with birth weight.

## Materials and Methods

### Design and Study Population

We performed a comparative, cross-sectional, and descriptive study. Samples from women without metabolic or chronic diseases before or during pregnancy from a previous cohort, recruited from the General Hospital of Leon, Guanajuato, Mexico, were analyzed. (Lazo-de-la-Vega-Monroy et al., 2020). The study was approved by the ethics committees of the University of Guanajuato and the corresponding health institutions (HGL-GTSSA002101-337 and CIBIUG P-23-2015 and P-43-2017). Women were recruited at the moment of delivery, and the informed consent was accepted before sample collection. Clinical and anthropometrical data were obtained from medical records and direct interview with the participants. For the neonates, anthropometrical measurements were performed according to the hospital’s standardized procedures by trained health staff and were taken from the medical record.

Only samples from pregnant women between 18 and 35 years old at term (>36 weeks of gestation), with singleton pregnancies and without diabetes mellitus, gestational diabetes, hypertension, antiphospholipid antibody syndrome, preeclampsia, alcoholism, smoking, IUGR diagnosis, or fetal distress, were included.

The gestational age was determined by the Capurro method, and neonates were classified according to national birth weight tables adjusted for gestational age and sex (Flores-Huerta and Martínez-Salgado, 2012; Norma Oficial Mexicana NOM-007-SSA2-1993, 1993). A total of 51 samples were divided according to birth weight into the following groups: SGA (small for gestational age) n = 14, AGA (adequate for gestational age) n = 18, LGA (large for gestational age) n = 10, and LGA-OB (large for gestational age products of mothers with obesity) n = 9. Clinical and anthropometric data, as well as the plasma from women and cord blood, were analyzed for the selected samples.

### Sampling Methods

Maternal blood samples were obtained during labor within 3–8 h of previous fasting. Placental and umbilical cord blood samples were collected immediately after delivery. A placental tissue of 5 × 5 was taken halfway between the cord insertion and the placental border within 30 min after delivery and was frozen in dry ice until use. Serum of the maternal peripheral blood and arterial umbilical cord blood samples was centrifuged, aliquoted, and stored at −20°C.

### Biochemical and Hormonal Assays

Biochemical variables in mother and umbilical cord blood (glucose and lipid profile) were measured by colorimetric enzymatic methodology (Spinreact). Maternal HbA1c detection was performed by using a cation exchange resin kit (Eagle diagnostics). Neonatal and maternal insulin concentrations were measured using an ultra-sensitive ELISA Kit (ALPCO). The HOMA IR Index was calculated by the following formula: Fasting insulin (μU/ml) × fasting glucose (mmol/L)/22.5.

### Placental Nutrient Transporters Protein Expression

Approximately, 150 mg of the placental tissue was taken for subsequent protein extraction with 300 µl of lysis buffer, which contained 30 µl of the protease inhibitor (MC Roche), 100 mM of Tris-HCL, 100 mM of NaF, 1 mM of disodium EDTA, 250 mM of saccharose, 150 mM of NaCl, 10 mM sodium orthovanadate, 10 mM NaPPi, and NP-40 at 1%. The samples were homogenized with a polytron and were incubated on ice for 2 h. The extracts were homogenized with a vortex for 30 min and centrifugated at 10,000 × *g* for 15 min at 4°C. The supernatant was collected, and the protein concentration for each homogenate was measured by the Lowry method using a BSA standard curve. Protein separation was performed by electrophoresis in 10% SDS-PAGE gels for GLUT1, GLUT3, SNAT1, SNAT4, and tubulin proteins. SDS-PAGE gels at 8% for SNAT2, FATP1, and FATP4 proteins were used. Proteins were transferred to nitrocellulose membranes (Hybond C Super, Amersham Pharmacia Biotech). The membranes were blocked with a 5% skim milk solution during 2 h and incubated overnight at 4°C with primary antibodies as follows: GLUT1 (Abcam ab-137656) 1:1500, GLUT3 (Santa Cruz Biotechnology Inc B-6; sc-74497) 1:500, SNAT1 (Santa Cruz Biotechnology Inc H-9; sc-137032) 1:2000, SNAT2 (Santa Cruz Biotechnology Inc H-60; sc-67081) 1:500, SNAT4 (Santa Cruz Biotechnology In N-19; sc-3344) 1:1000, FATP1 (Abcam ab-81875) 1:2000, FATP4 (Santa Cruz Biotechnology Inc B-5; sc-10), and tubulin (Sigma T6074) 1:8000. Then, membranes were washed and incubated with the corresponding anti-rabbit, anti-mouse, or anti-goat secondary antibody for each case, using 1:5000 to 1:200000 dilutions during 2 h at 4°C. The membranes were revealed using the Western ECL Substrate and ChemiDoc™ Touch Imaging System (Bio-Rad). Each band of interest was quantified by densitometry using Image-Lab Software 3.0 and normalized to the tubulin expression. Each sample was run in triplicates in different experiments.

### Placental Triglyceride Content

Placental triglycerides were quantified by an enzymatic-colorimetric assay. Briefly, 200 mg of the placental tissue was washed in cold PBS, resuspended, and homogenized in 1 ml of 5%NP-40/ddH2O solution using a Polytron PT 1200E homogenizer (Kinematica AG). Samples were heated at 80–100°C in a water bath for 2–5 min or until the NP-40 became cloudy and then cooled down to room temperature. Heating was repeated one more time to solubilize all triglycerides. Samples were centrifuged for 2 min at top speed using a microcentrifuge to remove any insoluble material and diluted 1:5 with ddH2O before proceeding with a commercial triglyceride assay (Spinreact). Results were normalized to the total protein concentration in each sample. (Schwartz and Wolins, 2007).

### Maternal Fatty Acid Profile

Maternal serum samples were treated for the quantification of fatty acids according to De La Rocha et al. (2016). Briefly, the sera were lyophilized, and 1 ml of the 0.5 M NaOH solution was added to each sample. Then, 10 µl of the internal standard (methyl pentacosanoate, Sigma-Aldrich) was added at 3 mg/ml, and samples were incubated at 90°C for 1 h in a water bath. The samples were allowed to cool at room temperature for half an hour, then 1.5 ml of boron trifluoride and methanol (Sigma Aldrich) were added, and incubated at 90°C for 30 min. Next, 3 ml of hexane was added, and each sample was vortexed for 2 min at medium speed, centrifuged for 5 min at 3,000 rpm at 4°C, and the organic phase was recovered, in clean conical tubes. The samples were stored at 4°C until use.

Before analyzing the samples, the hexane from the organic phase was evaporated to dryness with nitrogen gas, and the pellet was resuspended with 400 µl of isooctane (2,2,4-Trimethylpentane, Sigma-Aldrich) and transferred to a gas chromatography vial.

The samples were analyzed using a gas chromatograph (Perkin Elmer Clarus 680-5Q8), with a capillary column (Elitewax 10: 30 m × 0.25 mm ID 0.24 µm) coupled to a flame ionization detector (GC-FID). The programmed oven temperature ramp was as follows: initial temperature 40°C for 1 min increasing at a rate of 10°C/min up to 150°C, maintaining 1 min, and the final temperature was 230°C increasing at a rate of 5°C/min holding 20 min. The injector temperature was kept constant at 250 °C. Helium was used as the carrier gas at a flow rate of 2 ml/min. The sample was injected at a ratio of 10:1 split. TotalChrom Navigator-Clarus software was used for the data analysis. Fatty acid identification was made by comparing the retention times with the standards (Sigma-Aldrich) of each of the fatty acids of interest (palmitic, arachidonic, linoleic, and docosahexaenoic (DHA) acids) and normalized to the percentage of total fatty acids in the sample.

### Statistical Analysis

Data distribution was assessed by the Kolmogorov–Smirnov Test. Clinical data are presented with mean ± SD or median and interquartile range, according to distribution data. Differences between each experimental group were evaluated by ANOVA or Kruskal–Wallis tests. Spearman coefficients were used to evaluate correlations, and the multiple linear regression was performed to confirm associations. *p values* less than 0.05 were considered statistically significant. StatView statistical analysis software V.4.5 (Abacus Concepts, Berkeley, CA, United States) was used.

## Results

### Anthropometric, Clinical, and Biochemical Data

Anthropometric, clinical, and biochemical data of mothers and newborns are detailed in Table 1. The pregestational BMI of mothers from the LGA group was higher than that of mothers of SGA newborns (*p* < 0.05). LGA-OB mothers presented higher pregestational BMI in comparison with mothers of AGA (*p* < 0.05) and SGA newborns (*p* < 0.05). Gestational weight gain was not different between the groups, even after adjusting for weeks of gestation (*p* = 0.929, data not shown). No other differences between groups were observed in the metabolic profile of mothers.

**TABLE 1:** Clinical, biochemical, and anthropometric characteristics of mothers and their SGA, AGA, and LGA newborns.

	SGA (n = 14)	AGA (n = 18)	LGA (n = 10)	LGA-OB (n = 9)	*p*-value
**Maternal characteristics**					
Age (years)	22 (20–28)	21 (20–29)	27 (23–31)	25 (21–32)	0.250
Pregestational BMI (kg/m^2^)	22.7 ± 2.9	23.9 ± 3.2	25.4 ± 2.8^**+**^	31.3 ± 1.3*^+^	**<0.001**
Gestational weight gain (kg)	11.1 ± 4.4	13.5 ± 4.6	13.8 ± 4.5	12.8 ± 2.7	0.412
Blood glucose (mg/dl)	73.6 ± 14.8	82.6 ± 11.1	75.6 ± 13.7	78.8 ± 16.2	0.292
HbA1c (%)	5.2 (4.8-5.9)	5.5 (5.3-5.7)	5.9 (5.4-6.0)	5.6 (4.8-6.1)	0.489
Triglycerides (mg/dl)	177 (155-220)	225 (198-265)	217 (159-302)	215 (173-299)	0.265
Total cholesterol (mg/dl)	192 ± 36	209 ± 30	198 ± 41	210 ± 51	0.541
Insulin (uUI/l)	8.4 (5.0-26.2)	9.7 (6.0-13.9)	19.4 (7.7-30.9)	9.6 (7.3-29.0)	0.412
**Neonatal characteristics**					
Baby’s gender (male/female)	6/8	11/7	7/3	4/5	0.191
Delivery method (vaginal/c-section)	7/7	12/6	6/4	2/7	0.170
Gestational age (wk)	38.4 ± 1.1	38.7 ± 1.1	39.8 ± 0.9* ^**+**^	38.9 ± 0.6	**0.012**
Placental weight (g)	404.9 ± 64.2 *	608.7 ± 112.0	730.1 ± 80.3* ^**+**^	740.1 ± 119.8* ^**+**^	**<0.001**
Birth weight (g)	2,307 (2,143.8-2,407.8)*	3,272.5 (2,925.0-3,336.3)	3,915 (3,787.5-4,102.5) * ^**+**^	3,893 (3,815-4,127.5)* ^**+**^	**<0.001**
Birth lenght (cm)	45.6 ± 2.3*	50.9 ± 2.1	52.8 ± 2.1* ^**+**^	52.2 ± 2.1^**+**^	**<0.001**
Head circumference (cm)	32.2 ± 1.7*	34.6 ± 1.5	36.4 ± 1.6* ^**+**^	36.3 ± 0.7* ^**+**^	**<0.001**
Thoracic circumference (cm)	29.7 ± 1.1*	33.6 ± 1.6	35.8 ± 1.2* ^**+**^	35.2 ± 1.0* ^**+**^	**<0.001**
Abdominal circumference (cm)	27.3 ± 1.4*	30.8 ± 1.8	34.1 ± 2.2* ^**+**^	33.9 ± 2.2* ^**+**^	**<0.001**
Ponderal index (g/cm^3^)	2.52 (1.84-2.88)	2.42 (2.23-2.55)	2.71 (2.5-2.88)	2.75 (2.60-3.06) *	**0.004**
Triglycerides (mg/dl)	53.6 (36.3-71.4)	58.4 (54.0-66.6)	49.0 (45.0-58.8)	44.9 (38.0-52.85)	0.090
Total cholesterol (mg/dl)	69.9 (59.8-86.2)	83.7 (75.9-95.4)	76.5 (70.3-79.4)	65.4 (59.5-84.1)	0.053
HDL (mg/dl)	41.02 ± 7.6*	47.6 ± 7.1	42.2 ± 10.5	41.6 ± 6.4	0.087
LDL (mg/dl)	16.2 (11.4-30.8)	25.5 (10.0-34.1)	22.2 (11.7-29.5)	20.8 (11.1-30.1)	0.434
Glucose (mg/dl)	63.7 (45.1-85.1)*	77.1 (66.5-90.2)	61.8 (56.7-70.5)	63.5 (57.5-74.4)	0.07
Insulin (uUI/l)	1.6 (0.8-2.9)*	3.5 (2.4-4.9)	4.1 (3.5-5.3) ^**+**^	4.1 (1.4-7.7)^**+**^	**0.003**

Mean ± SD; median (25–75% quartile range); *p < 0.05 compared to control AGA, ^**+**^ *p*< 0.05 compared to SGA.

Anthropometric parameters in SGA, LGA, and LGA-OB newborns were different compared with AGA newborns. Birth weight, birth length, head circumference, thoracic circumference, and abdominal circumference were lower in SGA newborns (*p* > 0.05) and higher in LGA and LGA-OB newborns than AGA. Interestingly, only LGA-OB newborns differed in their ponderal index, being higher than the AGA group.

Cord blood HDL cholesterol, glucose, and insulin were 13.8, 17.4, and 54.3% lower in SGA, respectively, compared to AGA. In addition, cord blood insulin of LGA and LGA-OB was 17.1% higher than in SGA newborns. Cord blood triglycerides, total cholesterol, and LDL cholesterol were similar between the groups.

### Placental Expression of Glucose Transporters

**FIGURE 1:**
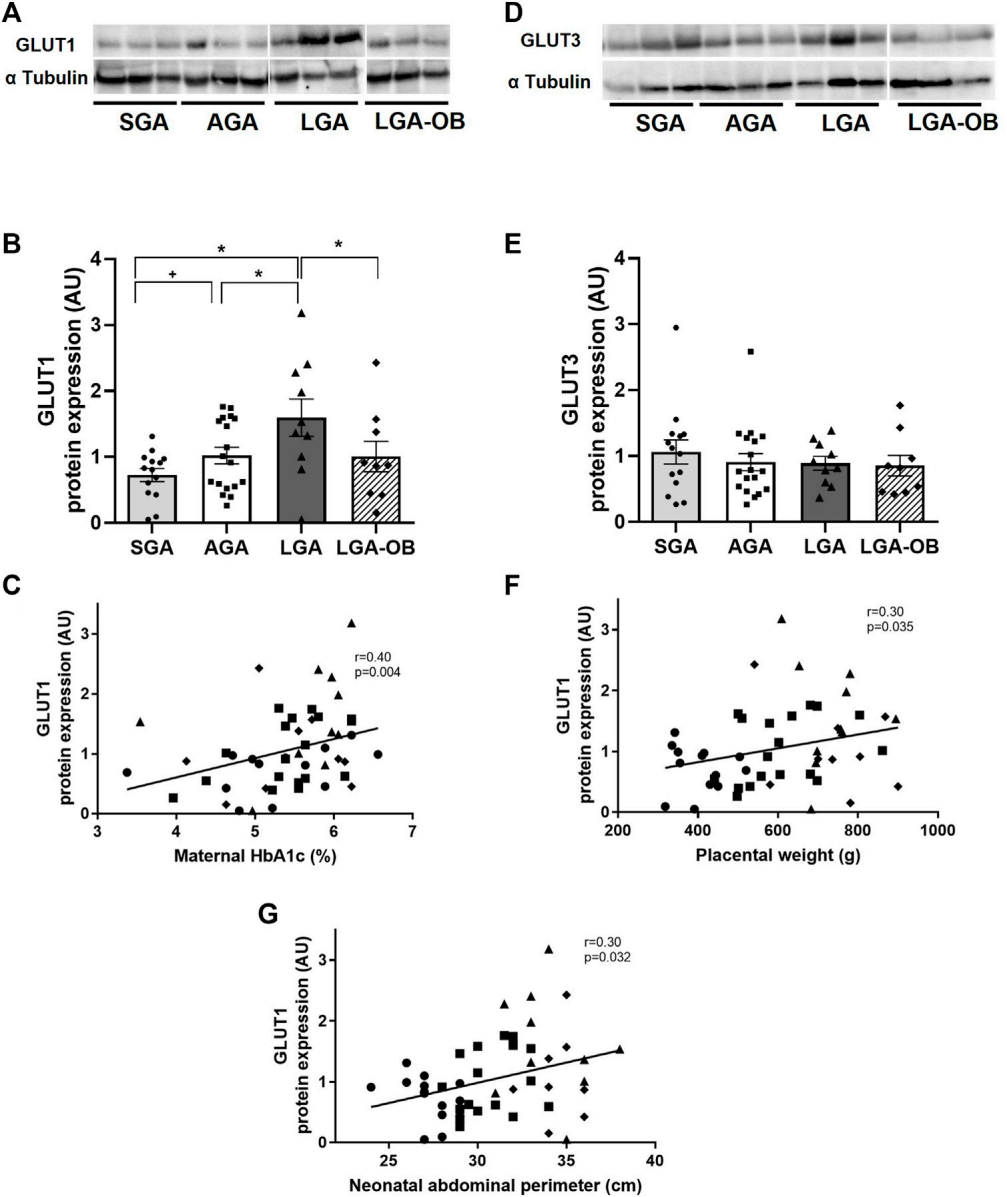
Protein expression of glucose transporters in placentas of SGA, AGA, LGA, and LGA-OB newborns. Representative Western blot of GLUT1 **(A)** and GLUT3 **(D)** in placentas of SGA (n = 14), AGA (n = 18), LGA (n = 10), and LGA-OB (n = 9) newborns. Bands presented correspond to the same membrane. Quantification of GLUT1 **(B)** and GLUT3 **(E)** protein expressions in the placenta by immunodetection. Data are mean ± SE. * denotes significant difference (*p* < 0.05) between groups by ANOVA. + denotes significant difference when compared to control AGA by Student´s *t* test. Correlation between the placental expression of GLUT1 with maternal HbA1c **(C)**, placental weight **(F),** and neonatal abdominal perimeter **(G)**.

The protein expression of the placental glucose transporter GLUT1 was higher in LGA and lower in SGA than the AGA group (Figures 1A,B). The GLUT1 expression in the LGA-OB group was similar to that of AGA but lower than the LGA group (Figures 1A,B). GLUT1 positively correlated with maternal HbA1c, placental weight, and neonatal abdominal perimeter (Figures 1C, F, and G, respectively). GLUT1 also correlated with the ponderal index (r = 0.3 and *p* = 0.032). The placental expression of GLUT3 did not differ between groups (Figures 1D,E). Both GLUT1 and GLUT3 expressions were similar between male and female newborns (*p* = 0.635 and 0.102, respectively).

### Placental Expression of Amino Acid Transporters

**FIGURE 2:**
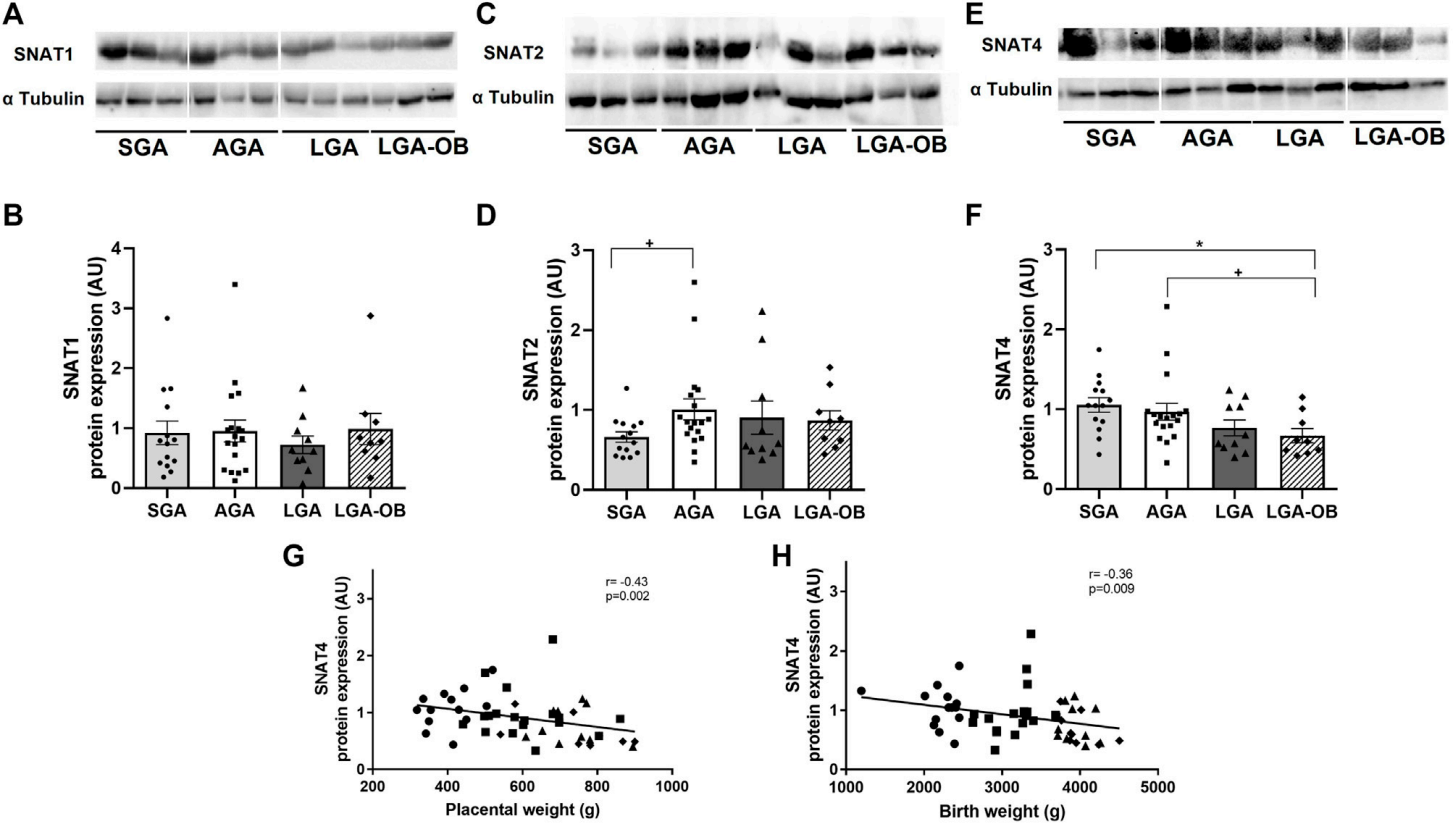
Protein expression of amino acid transporters in placentas of SGA, AGA, LGA, and LGA-OB newborns. Representative Western blot of SNAT1 **(A),** SNAT2 **(C),** and SNAT4 **(E)** in placentas of SGA (n = 14), AGA (n = 18), LGA (n = 10), and LGA-OB (n = 9) newborns. Bands presented correspond to the same membrane. Quantification of SNAT1 **(B),** SNAT2 **(D),** and SNAT4 **(F)** protein expressions in the placenta by immunodetection. Data are mean ± SE. * denotes significant difference (*p* < 0.05) between groups by ANOVA. + denotes significant difference when compared to control AGA by Student´s *t* test. Correlation between the placental expression of SNAT4 with placental weight **(G)** and birth weight **(H)**.

The placental expression of amino acid transporters SNAT1, SNAT2, and SNAT4 was evaluated. The SNAT1 expression showed no differences between SGA, AGA, LGA, and LGA-OB (Figures 2A,B), or either male or female placentas (*p* = 0.323) and did not correlate with any other maternal or neonatal variables. The protein expression of SNAT 2 was lower in SGA than that in AGA (Figures 2C,D). Placental SNAT4 was lower only in LGA-OB than that in AGA and SGA (Figures 2E,F). SNAT4 also correlated with placental weight (Figure 2G), birth weight (Figure 2H), and most neonatal anthropometrical variables such as size at birth (r = −0.289 and *p* = 0.04), thoracic perimeter (r = −0.31 and *p* = 0.03), and abdominal perimeter (r = −0.39 and *p* = 0.004). We found a positive correlation between SNAT1 and SNAT4 protein expressions (r = 0.297 and *p* = 0.035). No differences by neonatal sex were found for these transporters (*p* = 0.422 for SNAT2 and *p* = 0.084 for SNAT4).

### Placental Expression of Fatty Acid Transporters

**FIGURE 3:**
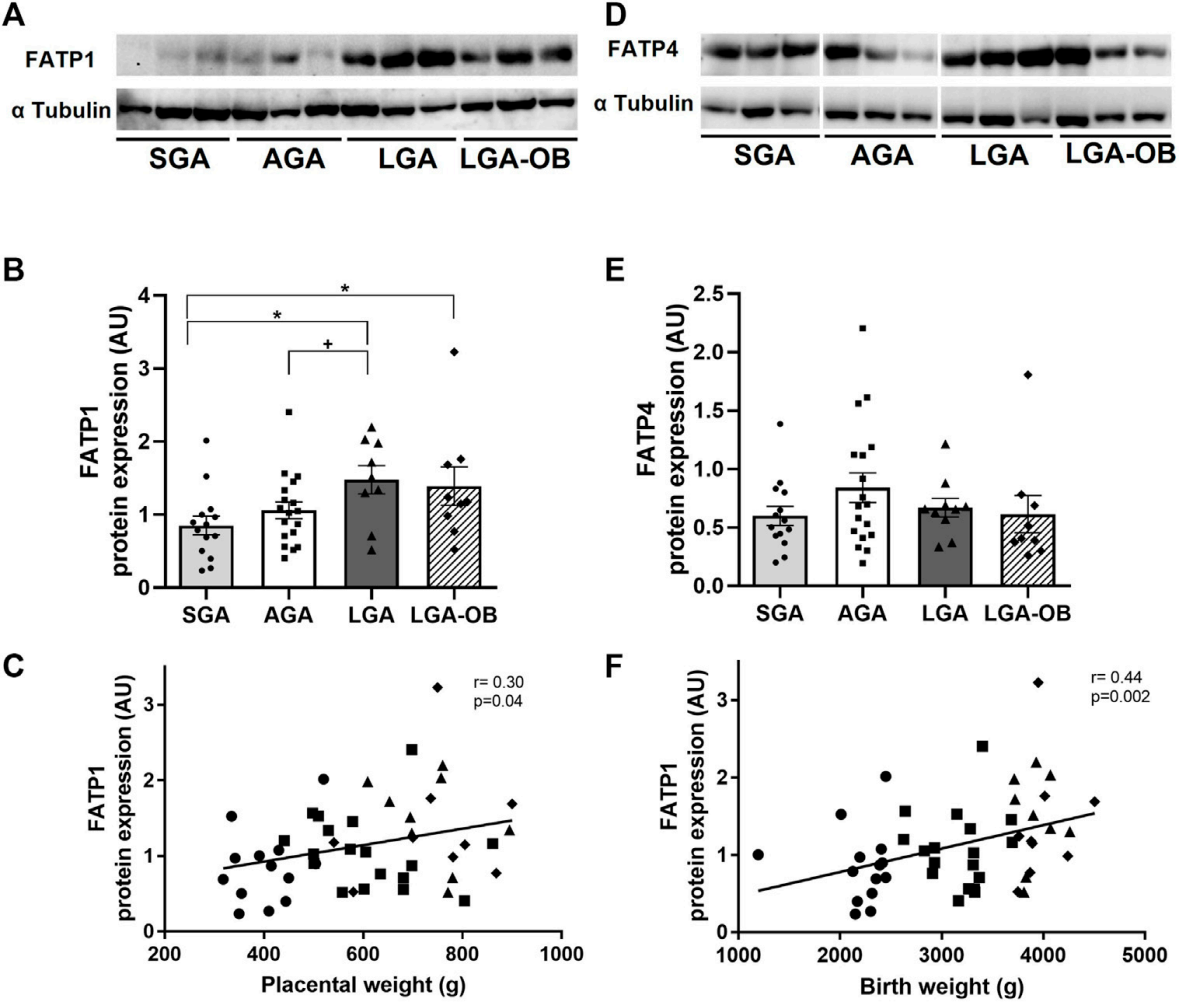
Protein expression of fatty acid transporters in placentas of SGA, AGA, LGA, and LGA-OB newborns. Representative Western blot of FATP1 **(A)** and FATP4 **(D)** in placentas of SGA (n = 14), AGA (n = 18), LGA (n = 10), and LGA-OB (n = 9) newborns. Bands presented correspond to the same membrane. Quantification of FATP1 **(B)** and FATP4 **(E)** protein expressions in the placenta by immunodetection. Data are mean ± SE. * denotes significant difference (*p* < 0.05) between groups by ANOVA. + denotes significant difference when compared to control AGA by Student´s *t* test. Correlation between the placental expression of FATP1 with placental weight **(C)** and birth weight **(F)**.

The fatty acid transporter FATP1 was lower in SGA placentas than in LGA and LGA-OB groups, while only LGA was higher than AGA (Figures 3A,B). The FATP1 expression positively correlated with placental weight (Figure 3C) and birth anthropometry, including birth weight (Figure 3F), size at birth (r = 0.436 and *p* = 0.001), cephalic perimeter (r = 0.28 and *p* = 0.05), thoracic perimeter (r = 0.58 and *p* = 0.006), and abdominal perimeter (r = 0.442 and *p* = 0.001). The placental FAPT4 expression was not different according to birth weight groups (Figures 3D,E). Placentas from male and female newborns showed similar FATP1 and FATP4 expressions (*p* = 0.656 and 0.528, respectively).

### Placental Triglyceride Content

**FIGURE 4:**
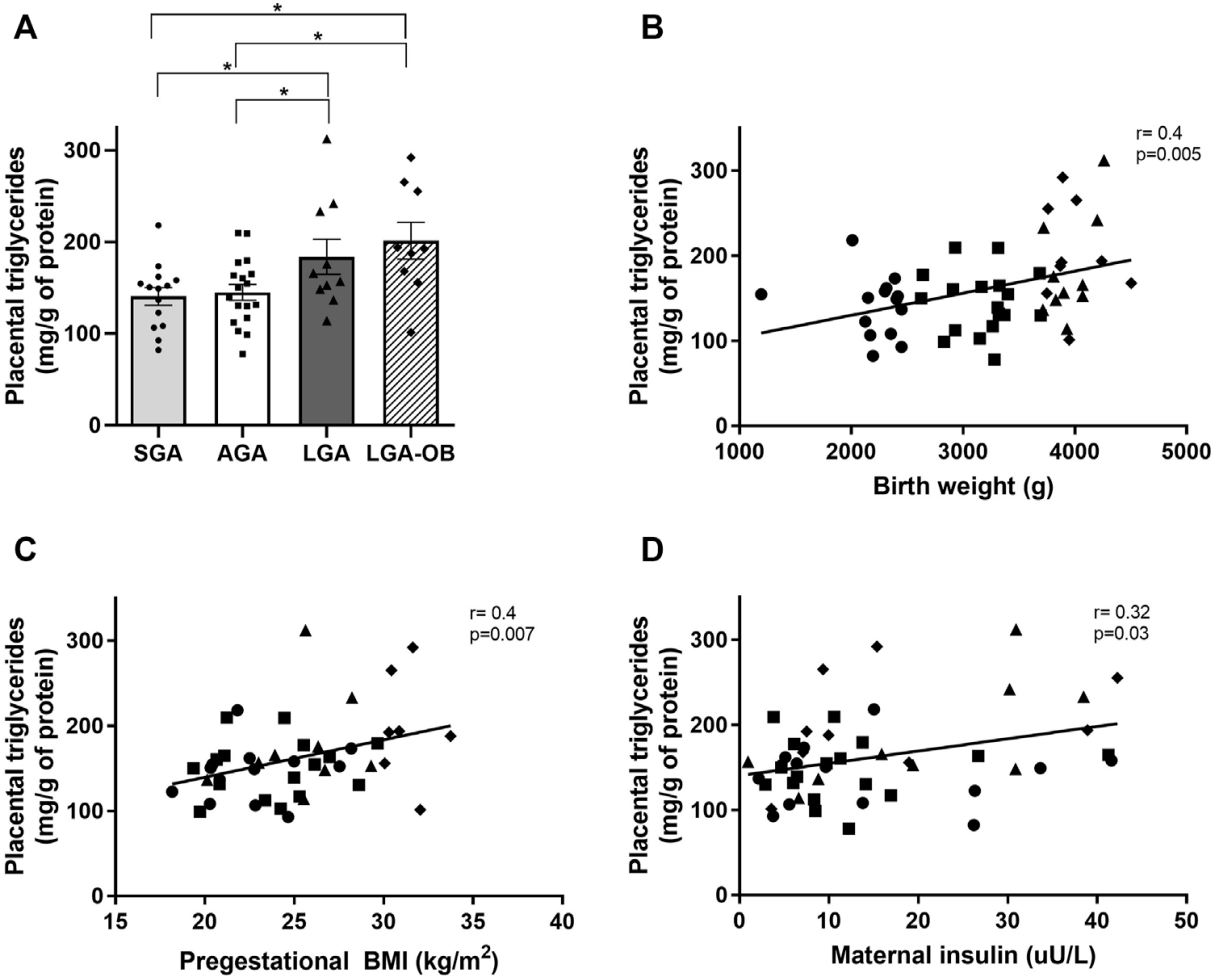
Triglyceride content in placentas of SGA, AGA, LGA, and LGA-OB newborns and correlation with neonatal and maternal variables. **(A)** Quantification of triglycerides in placentas of SGA (n = 14), AGA (n = 18), LGA (n = 10), and LGA-OB (n = 9) newborns by enzymatic-colorimetric method. Results were normalized to total protein in each sample. * denotes significant difference (*p* < 0.05) between groups by ANOVA. Correlation between placental triglycerides with birth weight **(B)**, pregestational BMI **(C),** and maternal insulin **(D)**.

We assessed the placental triglyceride content between the different birthweight groups. Placental triglycerides were elevated both in LGA and LGA-OB compared to AGA and SGA (Figure 4A), with no differences between sexes. The triglyceride content correlated positively with pregestational BMI, maternal insulin (Figures 4C,D), and maternal HOMA-IR (r = −0.302 and *p* = 0.033), together with birth weight (Figure 4B), size at birth (r = 0.32 and *p* = 0.021), cephalic perimeter (r = 0.33 and *p* = 0.02), thoracic perimeter (r = 0.37 and *p* = 0.007), and abdominal perimeter (r = 0.35 and *p* = 0.01) of newborns.

### Maternal Fatty Acid Profile

**FIGURE 5:**
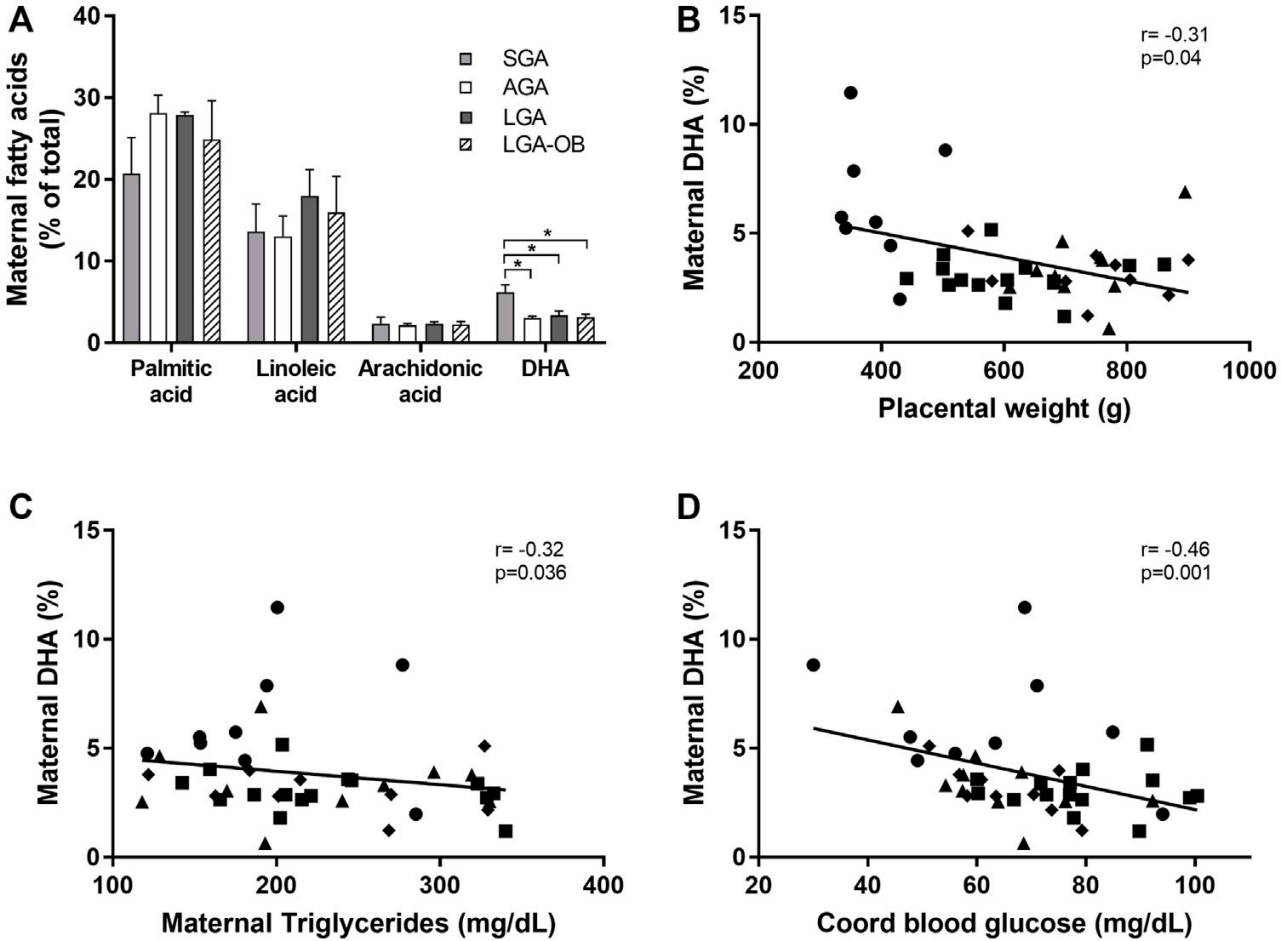
Maternal serum fatty acid profile and correlation of maternal DHA with maternal and neonatal variables. Maternal serum fatty acids **(A)** evaluated by gas chromatography. Fatty acid identification was made by comparing the retention times with the standards of each of the fatty acids of interest (palmitic, arachidonic, linoleic, and DHA acids) and normalized to the percentage of total fatty acids in the sample. * denotes significant difference (*p* < 0.05) between groups by ANOVA. Correlation between maternal DHA with placental weight **(B)**, maternal triglycerides **(C),** and cord blood glucose **(D)**.

The percentages of maternal palmitic (PA), linoleic (LA), and arachidonic acids (AA) were no different from birth weight classification, while DHA in mothers from SGA newborns was higher than that in AGA and the other groups (Figure 5A), with this difference due to a higher DHA concentration in mothers from males than in females (7.62 ± 0.69 and 4.43 ± 0.77, respectively, *p* = 0.004). Maternal DHA did not differ between males and females in AGA, LGA, or LGA-OB, nor were other differences between sexes found for the rest of the fatty acids (data not shown).

We found a negative correlation between maternal DHA and placental weight, maternal serum triglycerides, cord blood glucose (Figures 2B–D), and neonatal abdominal perimeter (r = −0.353 and *p* = 0.02). Although no differences in the other fatty acids were observed, palmitic acid correlated negatively with placental FATP4 (r = −0.384 and *p* = 0.01) and positively with cord blood insulin (r = 0.314 and *p* = 0.04). Linoleic acid correlated with palmitic acid (r = 0.488 and *p* = 0.001) and maternal cholesterol (r = −0.36 and *p* = 0.02). Arachidonic acid was inversely correlated with maternal triglycerides (r = −0.38 and *p* = 0.026) and directly with the FATP4 expression in the placenta (r = 0.34 and *p* = 0.045).

When performing a multivariate analysis, maternal DHA remained significantly associated with birth weight (t = −2.497, *p* = 0.018) and placental weight (t = −2.245, *p* = 0.032), independently of pregestational BMI, pregnancy weight gain, neonatal sex, and gestational age.

## Discussion

Adverse factors in early life result in fetal metabolic programming and increased risk of adult diseases, being birth weight an indirect marker of this unfavorable environment. The placenta plays a crucial role in modulating fetal growth not only by transporting nutrients but also by adapting its metabolism and function in response to maternal nutrition throughout pregnancy (James-Allan et al., 2020), thus acting as a nutrient sensor (Jansson and Powell, 2006). Most studies about placental nutrient transport are conducted in the context of pregnancy pathologies, but information on healthy or obese women considering neonatal outcomes, such as birth weight and metabolism, is lacking. In the present study, we evaluated the expression of several placental nutrient transporters in idiopathic birth weight alterations (SGA, AGA, and LGA) and maternal obesity (LGA-OB) in relationship to the maternal metabolic status and neonatal outcomes.

Glucose is the main energy substrate for fetal and placental growth. The fetus depends on maternal glucose availability, which is transported by placental GLUTs (Joshi et al., 2021). GLUT1 (Illsley, 2000) is the main placental glucose transporter, and although it is located primarily in the microvillous membrane (MVM), facing the maternal side of the placenta, the GLUT1 expression in the basal membrane (BM) facing the fetal capillaries is suggested as the rate limiting step for fetal glucose transfer (Jansson et al., 1993; James-Allan et al., 2019).

In the present study, an increased protein expression of GLUT1 was found in term placentas from LGA babies, but not in LGA-OB, compared to AGA. The overexpression of the GLUT1 transporter has been reported in placentas from mothers with type I diabetes (Jansson et al., 1999) and gestational diabetes [reviewed in Joshi et al. (2021)] although other studies have rendered discordant results (Jansson et al., 2001). Similarly, studies in placentas from women with obesity giving birth to normal weight babies have found no changes in the GLUT1 expression. However, a lower expression in the MVM and a higher expression in the BM have been observed from women with obesity delivering macrosomic newborns (James-Allan et al., 2019), which is consistent with the lack of changes we see in LGA-OB placental samples, comprising both maternal and fetal sides. Few studies have measured GLUT1 in the context of fetal growth restriction, and most of them report no alteration in GLUT1 (Joshi et al., 2021). In a recent study, a decrease of GLUT1 has been showed in IUGR, but not in SGA or macrosomic placentas, contrary to our findings (Stanirowski et al., 2021). These discrepancies may be explained by methodological reasons (immunohistochemistry vs. Western blot).

Several investigations suggest that regulation of the GLUT1 expression occurs through metabolic control and maternal glucose concentrations (Jansson et al., 1999; Joshi et al., 2021). Although the maternal metabolic status did not differ between groups, we found a positive correlation of the GLUT1 expression with maternal HbA1c, suggesting a possible role of the maternal glycemic status in GLUT1 regulation, even in the absence of diabetes mellitus.

It has been widely suggested that the placental GLUTs play a pivotal role in birth weight establishment and that their upregulation may lead to an increase in the glucose transport and fetal growth (Stanirowski et al., 2018; Joshi et al., 2021). The positive correlations between GLUT1 with the placental weight, ponderal index, and neonatal abdominal perimeter found in our study support this notion. Moreover, the altered placental GLUT1 expression is thought not only to regulate the glucose transfer but also to modulate the placental metabolic flexibility. Recently, studies in placental explants have shown that placental glucose dependency is positively correlated with the GLUT1 expression, while glucose flexibility is negatively associated with this transporter, but only in placentas from males (Wang et al., 2019). In contrast, our study found no differences in the GLUT1 expression between male and female placentas.

In opposition to the GLUT1 expression, no differences were found in the expression of GLUT3 in the placentas of the groups evaluated in the present study. While the syncytiotrophoblast GLUT3 expression both in the MVM and BM has been reported in humans (James-Allan et al., 2020), its expression and role in the placenta are less clear (Joshi et al., 2021). Previous studies refer no changes in the GLUT3 expression in gestational diabetes, IUGR, macrosomia (Kainulainen et al., 1997), or maternal obesity (James-Allan et al., 2020), while an increased expression in IUGR (Janzen et al., 2013; Stanirowski et al., 2021) and decreased expression in gestational diabetes (Zhang et al., 2016) but no changes in SGA (Stanirowski et al., 2021) have been reported in other studies. It is suggested that GLUT3’s function in the villous vessels at the maternal–fetal interface is the reuptake of glucose from the fetal circulation, increasing the glucose concentration in the placenta, possibly acting as an adaptive mechanism to protect the fetus from high glucose concentrations (Stanirowski et al., 2018). In this view, the lack of differences or correlation of the GLUT3 expression with maternal or neonatal variables in our study would be expected in the context of healthy normoglycemic mothers.

Placental amino acid transfer to the fetus is mediated by two coordinated systems, termed system A and L. System A transports nonessential neutral amino acids by a sodium-dependent mechanism, comprising the protein transporters SNAT1, SNAT2, and SNAT4, which are mainly located in the MVM (Vaughan et al., 2017). Studies in SNAT2- (Vaughan et al., 2021) or SNAT4 (Matoba et al., 2019)-knockout mice specifically in trophoblasts have shown that these two transporters are crucial for placental and fetal growth, while reduced placental amino acid transfer precedes the onset of IUGR in rat (Pantham et al., 2016) and primate (Rosario et al., 2021) models of maternal protein restriction.

In the present study, the SNAT1 expression showed no differences between birthweight groups. However, SNAT2 was lower in placentas from SGA newborns. These results resemble the findings in primate models of maternal nutrient restriction, in which a decrease in the capacity and activity of system A transport and in birth weight is found, together with a decreased placental expression of SNAT2, but normal SNAT1 (Kavitha et al., 2013). Also, protein (Rosario et al., 2011) or folate (Rosario et al., 2017) restriction during pregnancy decreases the expression of SNAT2 in mice. A study in human SGA from healthy mothers found a reduction in total placental system A amino acid transport activity, without measuring the SNAT expression (Shibata et al., 2008). In the human placenta from preterm and term IURG, lower SNAT1 (Chen et al., 2015) and SNAT2 (Mandò et al., 2013; Chen et al., 2015) protein expressions have been found. In the present study, the SNAT2 expression was similar between AGA, LGA, and LGA-OB, and no correlation of this transporter with birthweight was found. Conversely, the system A activity has been found increased in placentas from large babies of obese women, being SNAT2, but not other isoforms, correlated with birth weight and pregestational BMI, although the comparison between normal and obese BMI or AGA and LGA babies was not reported (Jansson et al., 2013).

The placental expression of SNAT4 was lower only in placentas of the LGA-OB group compared to AGA and SGA. Furthermore, a negative correlation was observed between this transporter and placental weight and birth anthropometry. The placental amino acid transport decreases in placentas of obese women with normal weight babies, potentially associated with maternal leptin resistance. Also, immunohistochemistry analysis has revealed a decrease in SNAT4, but not other isoforms, in these placentas (Farley et al., 2010).

Studies exploring the contribution of each SNAT isoform to amino acid transport in the normal human placenta have proposed that SNAT1 is the main mediator of α-methylaminoisobutyricacid (MeAIB) uptake in the MVM, accounting for approximately 70% of the transport, followed by 30% SNAT4 and 1% SNAT2 in humans. Nevertheless, the regulation of the expression of amino acid transporters could be mediated according to its specific substrate availability (Takahashi et al., 2017). SNAT4 has low affinity for MeAIB and greater affinity to cationic amino acids (arginine and lysine) (Hatanaka et al., 2001). The placenta increases system A activity depending on the type and concentrations of amino acids available in order to ensure the optimal fetal growth (Snook Parrott et al., 2007). Hence, the placental SNAT4 expression may serve as a compensatory mechanism, either due to a decrease (Belkacemi et al., 2011) or an increase in nutrient availability (Farley et al., 2010), or else, a saturation of SNAT1 and SNAT2 transporters (Takahashi et al., 2017), which is supported by the positive correlation between SNAT1 and SNAT4 found, and a lower SNAT4 expression in the placenta of obese mothers.

The role of FATPs in the human placenta and their regulation is poorly understood. In the present study, FATP1, but not FATP4, placental expression was positively correlated with placental weight and birth anthropometry, being lower in SGA and higher in both LGA and LGA-OB. Similarly, FATP1 mRNA has been correlated with maternal BMI in human placentas (Hirschmugl et al., 2017). On the contrary, FATP1 mRNA has found to be lower in placentas from women with overweight or obesity, together with high placental long-chain polyunsaturated fatty acids (LCPUFAs) and low saturated fatty acids (Segura et al., 2017). FATP1 transports long-chain fatty acids, and its gene expression in the placenta has been positively correlated with placental DHA in placental phospholipids and triglycerides and negatively with cord blood AA in phospholipids (Larqué et al., 2006). Moreover, the FATP1 expression is also associated with a greater uptake of palmitate and increased triglyceride accumulation when overexpressed in myotube C2C12 cells (Kawaguchi et al., 2014). Also, an increase in FATP1 mRNA has been show in cultured human trophoblast cells with PPARg and RXR agonists, suggesting that regulation of this transporter may also be dependent on the different types and concentrations of maternal circulating nutrients (Schaiff et al., 2005). Supporting this view, other nutrients have been associated with the FATP1 expression, such as vitamin B12, whose restriction in mice lowers its mRNA, possibly by epigenetic mechanisms (Wadhwani et al., 2013).

To further explore if changes in FATP1 protein could be related to placental lipid accumulation or nutrient availability, we measured total placental triglycerides (TG) and plasmatic fatty acid maternal profiles. The placental triglyceride content was higher in LGA and LGA-OB, similar to previous reports in women with obesity (Hirschmugl et al., 2017) but in contrast to other studies (Hulme et al., 2019), possibly due to the fact that birth weight groups were not taken into account in previous studies. Placental TG correlated positively with birth anthropometry and maternal pregestational BMI. Moreover, a correlation with maternal insulin and HOMA-IR was found, but not with FATP1 or FATP4, which is consistent with *in vitro* studies in the human placenta, where exposure to elevated glucose alone (Hulme et al., 2019) or in combination with high insulin exposure (Mishra et al., 2020) promotes placental TG accumulation without changing the FATP expression.

Maternal LCPUFAs, such as DHA and AA, are determinant for fetal growth and, particularly, for fetal brain development and postnatal neurodevelopment, as well as for the placental metabolism itself (Duttaroy and Basak, 2020). In the present study, total maternal DHA was negatively associated with birth weight and abdominal perimeter independently from other maternal factors, presumably through an effect of DHA on the placental weight or function. These results agree with a recent study, in which concentrations of maternal and umbilical DHA in different phospholipid subclasses were lower in obese mothers of male fetuses, but not females, despite an increase in the lysophosphatidylcholine-DHA transporter MFSD2a in BM from male placentas, suggesting an adaptative mechanism of DHA transport. Similarly, no changes in FATPs in either the MVM or BM were found (Powell et al., 2021). Our findings further expand these observations regarding the relevance of sexual dimorphism for placental function, particularly in lipid transfer and metabolism, as an increase in total maternal DHA in SGA, attributable to mothers from SGA males, was found, just as previously shown for maternal obesity (Powell et al., 2021). Indeed, an increasing body of evidence has demonstrated that placentas from females grow slower than placentas from males, and both differ in their adaptative responses to environmental factors (Kalisch-Smith et al., 2017), which emphasizes the importance of evaluating males and females separately in placenta studies.

One of the main strengths of the present study is the inclusion of placentas from both extremes of birth weight, allowing to compare these two opposite conditions in healthy pregnancy, which may pose an increased risk for those newborns in metabolic disease in the future life. In particular, by separating the LGA newborns by the presence or absence of pregestational obesity, differences in the nutrient transporter GLUT1, but not in amino acid or fatty acid transporters, could be detected. Moreover, despite a similar metabolic profile and ponderal weight gain compared to AGA newborns, both LGA and LGA-OB mothers present hyperinsulinemia, while the LGA from obese mothers may have increased adiposity, which is suggested by the higher ponderal index found only in this group. Together with our findings in placental TG and maternal fatty acids, we can conclude that LGA babies born from normal or obese mothers have altered placental adaptations, which can possibly be exacerbated by maternal adiposity and hormonal status.

The lack of precise nutritional information is indeed a limitation to the study. It has been described that a diet of high fat and sugar induces alterations in the expression and activity of placental nutritional transporters, which has negative consequences on the growth of the offspring (Rosario et al., 2015). Having this information would surely help in elucidating the mechanisms underlying the differences between the study groups, mostly for the fatty acid profile, but also for those transporters whose placental expression has been associated with maternal macro or micronutrients, such as SNAT2 (Matoba et al., 2019) (51) and FATP1 (Schaiff et al., 2005; Wadhwani et al., 2013).

Another limitation is that the transporter expression was measured at term in placental samples including both the MVM and BM together, and their activity was not evaluated. Thus, we cannot establish if the differences in the protein expression could account for modifications in glucose, amino acid, or fatty acid transport. Indeed, the protein expressions of SNAT2, SNAT4, and GLUT1 are higher in term placentas compared to early gestation ones, and FATP4 is lower. Also, system A amino acid transport is higher at term (James-Allan et al., 2020). Moreover, location of the transporters at the membrane also influences their activity. Particularly, downregulation of mTORC1 has been shown to modulate the ubiquitination of SNAT2 by increasing ubiquitin ligase Nedd4-2, which favors the proteosomal degradation of the protein, thus decreasing its expression in the plasma membrane (Chen et al., 2015; Rosario et al., 2016). In a recent study, we found that the mTOR expression and activation was positively associated with the placental weight and birth weight, while AMPK, an upstream kinase-inhibiting mTOR, correlated negatively with the birth weight in healthy mothers (Lazo-de-la-Vega-Monroy et al., 2020). Together, these results support the potential role of nutrient-sensing pathways in modulating the expression and activity placental transporters and thus fetal growth. More studies in normal and pathological human pregnancies integrating the expression, location, and activity of transporters’ isoforms and their regulation, either by hormonal or nutritional factors, will increase the knowledge in the mechanisms modulating the placental nutrient transport.

In conclusion, we found that the placental protein expression of specific nutrient transporters is related to placental weight establishment and fetal growth and may depend on maternal factors such as glycemia and pregestational BMI even in the absence of pregnancy disorders. Additionally, we reported differences in the placental TG content and maternal DHA status associated with birth weight, with sexual dimorphism in circulating maternal DHA of SGA mothers. Further studies evaluating the potential mechanisms for regulating the expression of these nutrient transporters, such as maternal micronutrients or placental energy sensors, will open the door for potential therapeutic targets, as well as for defining the quality and quantity of nutrients required for placental function and hence fetal growth and future metabolic diseases.

## Data Availability

The raw data supporting the conclusions of this article will be made available by the authors upon reasonable request.
